# Beyond Volume Metrics: Outcomes of Open Abdominal Aortic Aneurysm Repair in a Small-to-Medium Volume Hospital—Challenging the Centralization Paradigm: A Single-Centre Retrospective Analysis

**DOI:** 10.3390/jcm15155939

**Published:** 2026-07-30

**Authors:** Alessandro Robaldo, Pietro Federico Ricciardi, Luca Giovannacci, Alexandre Azoulay, Elena Garbero, Ludovica Ettorre, Jacopo Galafassi, Matteo Bernasconi, Giorgio Prouse

**Affiliations:** 1 Division of Vascular Surgery and Angiology, Centro Vascolare Ticino, Ente Ospedaliero Cantonale, 6900 Lugano, Switzerland; pietro.ricciardi@chuv.ch (P.F.R.); luca.giovannacci@eoc.ch (L.G.); afm.azoulay@gmail.com (A.A.); elena.garbero@eoc.ch (E.G.); ludovica.ettorre@eoc.ch (L.E.); jacopo.galafassi01@gmail.com (J.G.); matteo.bernasconi@eoc.ch (M.B.); giorgio.prouse@eoc.ch (G.P.); 2 Vascular Surgery Unit, Heart and Vessel Department, University Hospital of Lausanne (CHUV), 1011 Lausanne, Switzerland; 3 Department of Thoracic and Vascular Surgery, Arnaud de Villeneuve Hospital, 34090 Montpellier, France

**Keywords:** abdominal aortic aneurysm, open surgical repair, centralisation, surgeon volume, hospital volume, surgical outcomes, surgical teaching, vascular surgery

## Abstract

**Background/Objectives**: Centralisation of open abdominal aortic aneurysm (AAA) repair is widely advocated, but supporting evidence often conflates institutional volume with individual surgeon expertise. We report outcomes of open AAA repair at a small- to medium-volume centre and assess the contributions of surgeon experience, multidisciplinary perioperative care, and supervised surgical teaching. **Methods**: Retrospective analysis of all consecutive patients undergoing open AAA repair between January 2017 and December 2023. Endovascular procedures were excluded. The primary endpoint was 30-day all-cause mortality. Secondary endpoints included perioperative morbidity, acute kidney injury (AKI), length of stay, and overall survival. **Results**: A total of 195 patients underwent open AAA repair (162 elective; 33 urgent/emergency), with a mean of 27.9 cases annually. Mean age was 70.2 years (SD 8.1), and 85.6% were male. Thirty-day mortality was 1.5% overall (3/195) and 1.2% (2/162) for elective cases, with no significant difference between elective and urgent/emergency cases (1.2% vs. 3.0%; *p* = 0.430). No perioperative strokes occurred; myocardial infarction occurred in three patients (1.5%). Median operative time was 261 min (IQR 210–320), reflecting systematic supervised teaching in approximately 95% of cases. AKI occurred in 10.3%; clinically significant renal insufficiency at 30 days occurred in 5.1%, and no patient required permanent renal replacement therapy. The perioperative reintervention rate was 9.2%, and median hospital stay was 8 days (IQR 7–12). At a mean follow-up of 62.3 months (SD 25.1), overall mortality was 7.7% and aneurysm-related mortality 1.0%. Kaplan–Meier estimated overall survival was 98.5%, 95.8%, and 89.0% at 1, 3, and 7 years, respectively. **Conclusions**: Favourable outcomes can be achieved at small- to medium-volume centres through concentrated surgeon expertise, structured teaching, and a high-functioning multidisciplinary team. These findings support a more nuanced assessment of open AAA repair that considers surgeon experience, team performance, and demonstrated outcomes rather than institutional volume alone.

## 1. Introduction

Abdominal aortic aneurysm (AAA) remains a leading cause of cardiovascular mortality, with open surgical repair (OSR) representing the definitive treatment for anatomically complex cases, younger patients, and situations where endovascular repair is not feasible or durable [1,2]. Over the past two decades, the progressive expansion of endovascular aortic repair (EVAR) has reduced the volume of open aortic procedures at most centres, raising concerns about the maintenance of surgical competence [3,4], while contemporary evidence on EVAR durability, sac dynamics, and long-term reintervention has been consolidated in the current ESVS guidelines [1].

Professional societies—including the European Society for Vascular and Endovascular Surgery (ESVS) and the Society for Vascular Surgery (SVS)—have advocated for the centralisation of complex aortic procedures to high-volume institutions [1,2]. Registry-based analyses and systematic reviews of both open surgical repair and EVAR [5,6] have broadly supported this volume–outcome relationship, with the German nationwide study by Trenner et al. demonstrating significantly lower in-hospital mortality and complication rates at hospitals performing more than 30 AAA procedures per year and identifying a continuous-volume threshold of approximately 75 elective cases per year as optimal [4].

However, the centralisation paradigm contains an inherent conceptual weakness: it conflates institutional volume with the quality of the individuals and systems delivering care. Liang et al. demonstrated that both hospital and individual surgeon volume independently predict mortality after open AAA repair, with surgeon volume emerging as the primary driver; high-volume institutions cannot compensate for low-volume lead surgeons [7]. The reverse may also be true: experienced surgeons operating within a competent multidisciplinary team at a moderate-volume institution may achieve results equivalent to those of high-volume academic centres.

A further dimension rarely addressed in the volume–outcome literature is the role of supervised surgical training and the contribution of the entire perioperative team—anaesthesiologists, nursing staff, and intensive care specialists—to outcomes. Our centre integrates structured, directly supervised operative teaching and operates with a well-established multidisciplinary perioperative protocol. We hypothesize that this model supports favourable outcomes independent of institutional volume. The present study reports 195 consecutive open AAA repairs performed between January 2017 and December 2023. We benchmark our results against published high-volume centre standards and contribute to the evolving debate on the true determinants of clinical outcomes in open aortic surgery.

## 2. Materials and Methods

### 2.1. Study Design and Patient Selection

Data were prospectively collected in a dedicated institutional database (REDCap version 16.0.35); the study objectives and analysis were retrospectively defined. All consecutive patients who underwent open surgical repair of an AAA at our vascular surgery unit between January 2017 and December 2023 were included. Operative indications followed current ESVS guidelines [1]: elective repair was offered to male patients with infrarenal or juxtarenal diameter ≥ 55 mm and to female patients with diameter ≥ 50 mm; symptomatic or rapidly expanding aneurysms (>10 mm growth/year) were repaired regardless of diameter; ruptured AAA constituted an absolute indication for emergency intervention. Patients with aortic dissection as the primary pathology and those undergoing endovascular repair (EVAR, fenestrated EVAR, branched EVAR, or hybrid procedures) were excluded.

In the emergency setting, endovascular repair is the preferred approach for ruptured AAA at our institution whenever it is technically applicable. Open repair is undertaken when endovascular treatment is precluded, principally by unsuitable proximal neck or iliac anatomy, or when the clinical situation does not permit the anatomical assessment required for endovascular planning. The ruptured cases included in the present series therefore represent those ruptures in which endovascular repair was not feasible and not the totality of ruptured AAA managed at our centre during the study period. Furthermore, the institutional database from which this series is drawn was constructed around operated cases; patients with ruptured AAA who died in the emergency department or during inter-hospital transfer, and those turned down for surgical treatment on grounds of age, comorbidity, or moribund presentation, were not entered into it, and their number cannot be retrieved retrospectively. The denominator of the ruptured subgroup reported here is therefore restricted to patients who survived to reach the operating theatre and undergo aortic clamping.

During the COVID-19 pandemic (2020–2022), cantonal health authority directives restricted elective surgical activity. Annual case volumes nevertheless remained stable, reflecting a maintained urgent and symptomatic caseload.

### 2.2. Surgical Team, Teaching Programme, and Institutional Context

Our vascular surgery unit is a small- to medium-volume centre within a regional hospital in Switzerland. Open aortic reconstructions are performed by three senior vascular surgeons, each with more than 10 years of postgraduate training and independent practice at major academic vascular surgery centres. All cases—elective and emergency—are personally performed or directly supervised by one of these senior surgeons. The standard operating team comprises one senior surgeon, one chief resident and one senior registrar. Approximately 95% of cases include a training component, with chief residents and senior registrars performing defined operative phases under the immediate, hands-on supervision of the scrubbed senior surgeon. No procedure is delegated for independent trainee performance.

### 2.3. Anaesthetic Team and Perioperative Management

Our anaesthesiology department manages the full breadth of major surgical procedures at our institution, including major hepatic resections, pancreaticoduodenectomy, complex oncological surgery, and neurosurgical interventions, maintaining a high level of competence in haemodynamic management of major haemorrhage and complex coagulation. A designated anaesthesiologist with specific expertise in aortic and major vascular surgery is assigned to all elective complex aortic cases. Their responsibilities include invasive haemodynamic monitoring, goal-directed fluid therapy, management of aortic cross-clamping and unclamping haemodynamics, renal protection protocols, intraoperative coagulation management, and cell salvage management in collaboration with the postoperative intensive care team.

### 2.4. Surgical Technique

**Figure 1:**
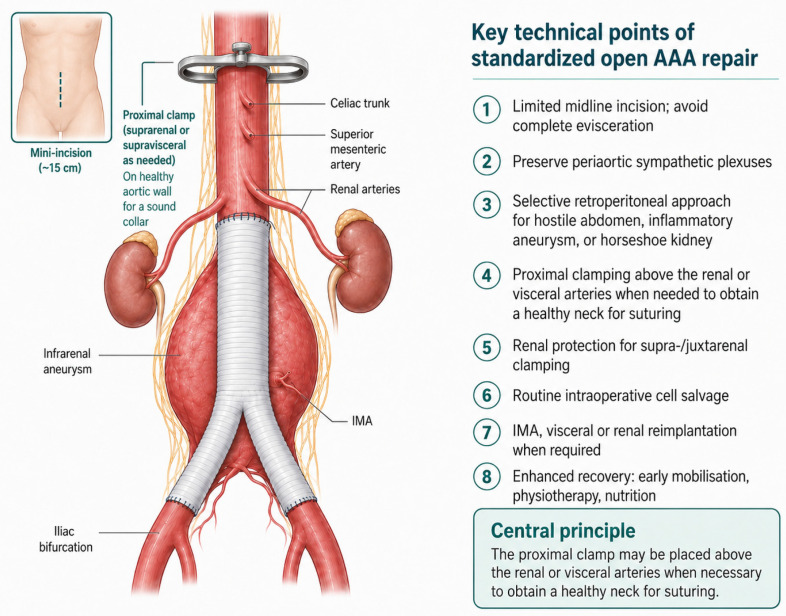
Key technical points of the standardised open AAA repair: (1) limited midline incision with avoidance of complete evisceration; (2) preservation of the periaortic sympathetic plexuses; (3) selective retroperitoneal approach for a hostile abdomen; (4) proximal clamping on healthy aortic wall to obtain a sound collar, irrespective of the final clamp level; (5) renal protection strategy for suprarenal or juxtarenal clamping; (6) routine intraoperative cell salvage; (7) reimplantation of the inferior mesenteric, visceral or renal arteries when required; and (8) a structured enhanced-recovery pathway. Not to anatomical scale.

Our technical approach is standardised and built on principles refined over decades of prior high-volume practice. Key elements include: (i) limited midline incision; (ii) avoidance of complete intestinal evisceration, with retraction using moist packs; (iii) preservation of the periaortic sympathetic plexuses; (iv) selective retroperitoneal (lombotomy) approach for hostile abdomen, severe obesity, horseshoe kidney, inflammatory AAA or difficult proximal neck; (v) systematic renal protection strategy for suprarenal or juxtarenal clamping (pre-operative hydration, intraoperative iv mannitol, selective cold renal perfusion, and minimisation of renal ischaemia time); (vi) routine intraoperative autologous blood transfusion (cell salvage) as standard institutional protocol in all non-infected cases; (vii) selective inferior mesenteric artery (IMA) reimplantation based on backflow assessment and Doppler evaluation of collateral circulation; and (viii) early postoperative mobilisation, chest physiotherapy, and early enteral nutrition as part of a structured enhanced recovery pathway. These technical elements are summarised schematically in Figure 1.

Endovascular aortic balloon occlusion (REBOA) has been progressively adopted at our institution over the study period as an adjunct to haemodynamic stabilisation in ruptured AAA and is currently employed to obtain temporary proximal control during induction and initial exposure when open repair is undertaken in the haemodynamically unstable patient. Its use was not analysed in the present series.

### 2.5. Outcomes and Statistical Analysis

The primary endpoint was 30-day all-cause mortality. Secondary endpoints included perioperative major adverse cardiovascular and cerebrovascular events (MACCE: stroke, myocardial infarction, cardiac failure/arrhythmia); pulmonary complications; acute kidney injury (AKI) by KDIGO criteria—defined as a serum creatinine increase of ≥26.5 μmol/L within 48 h, ≥1.5× baseline within 7 days, or urine output < 0.5 mL/kg/h for ≥6 h; colonic, limb, and spinal cord ischaemia; urinary complications; delirium; wound and prosthesis complications; reintervention; and overall survival at last follow-up.

Reinterventions were categorised as acute (within the perioperative admission) or elective late (planned procedures performed after discharge). Continuous variables are presented as mean ± SD and as median with interquartile range (IQR), as appropriate; categorical variables are presented as number (percentage). Comparisons between elective and urgent/emergency groups used the Mann–Whitney U test for continuous variables and Fisher’s exact test for categorical variables; *p* < 0.05 was considered statistically significant. Long-term overall survival was estimated using the Kaplan–Meier method, with follow-up censored at 31 December 2025. Statistical analyses were performed using SPSS version 27.0 (IBM Corp., Armonk, NY, USA).

### 2.6. Ethical Considerations

This study was conducted in accordance with the Declaration of Helsinki. As a retrospective analysis of routinely collected clinical data, the study was approved by the institutional ethics committee (approval number 204-00501 CE 4561) with waiver of formal written informed consent for retrospective anonymized data use. Patient consent was obtained for the use of pseudonymized clinical data for research purposes at the time of admission. A no-objection letter was sent to patients who had not previously given this research consent.

### 2.7. Generative Artificial Intelligence (GenAI)

GenAI was used to assist with language editing and improvement of manuscript readability. The authors reviewed and verified all content and remain fully responsible for the final manuscript.

## 3. Results

### 3.1. Annual Case Volume and Patient Demographics

**Figure 2:**
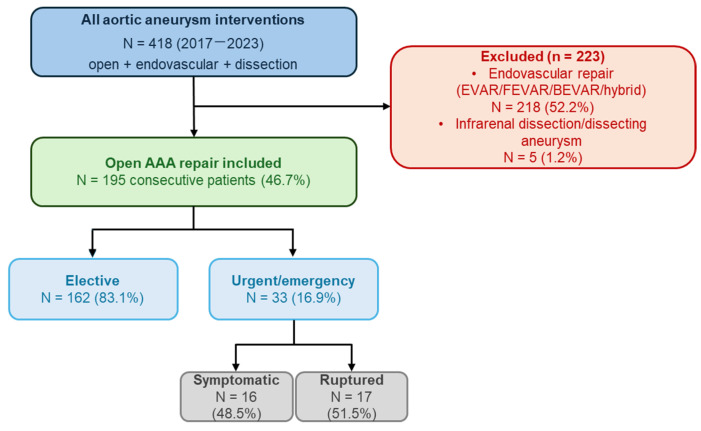
Study flow diagram. From all aortic aneurysm interventions performed during the study period, endovascular procedures (EVAR, FEVAR, BEVAR, and hybrid; *n* = 218, 52.2% of all aortic interventions) and cases with aortic dissection as the primary pathology (5 cases of infrarenal dissection or dissecting abdominal aneurysm) were excluded from the 418 abdominal aortic interventions, yielding 195 consecutive open AAA repairs. Cases were stratified by clinical presentation into elective (*n* = 162), symptomatic non-ruptured (*n* = 16), and ruptured (*n* = 17); the symptomatic and ruptured groups together constituted the urgent/emergency cohort (*n* = 33). Seven patients (3.6%) were lost to follow-up over the 7-year period.

Between January 2017 and December 2023, a total of 418 patients with abdominal aortic aneurysm were treated at our centre. Patients who underwent endovascular repair or presented with infrarenal dissection or a dissecting aneurysm were excluded from the analysis. The study included 195 consecutive patients that underwent open AAA repair. The patient selection and exclusion process is summarised in the study flow diagram (Figure 2). Annual case distribution for the study years 2017–2023 was 29 cases in 2017, 22 in 2018, 31 in 2019, 26 in 2020, 30 in 2021, 29 in 2022, and 28 in 2023, for a mean of 27.9 cases per year (range 22–31). The majority of patients were male (167/195, 85.6%), with a median age of 70 years (IQR 65–76; range 40–88) and mean BMI of 26.1 kg/m^2^ (SD 4.1).

The cohort represented a high-risk surgical population: hypertension was present in 72.8%, current or former smoking in 53.3%, coronary heart disease in 29.7%, COPD in 20.0%, diabetes in 18.5%, and congestive heart failure in 12.3%; previous open abdominal surgery had been performed in 14.9% of patients. ASA physical status was class II in 17.4%, class III in 66.2%, and class IV in 15.9%. Regarding renal function, 25.4% had CKD stage 1, 71.8% stage 2, 2.1% stage 4, and 0.7% stage 5 (Table 1).

### 3.2. Aneurysm Characteristics and Operative Data

Median aneurysm diameter was 56 mm (IQR 54–62; range 33–120). Aneurysms were asymptomatic in 162 cases (83.1%), symptomatic in 16 (8.2%), and ruptured in 17 (8.7%); fusiform morphology was recorded in 71.3%.

Of 195 patients, 162 (83.1%) underwent elective repair and 33 (16.9%) underwent urgent or emergency repair. The transperitoneal approach was used in 184 cases (94.4%) and the retroperitoneal approach in 11 (5.6%). Aortic clamping was infrarenal in 128 cases (65.6%), transrenal in 24 (12.3%), and suprarenal in 43 (22.1%). Reconstruction comprised straight tube grafts in 97 cases (49.7%), aorto-bi-iliac bifurcated grafts in 76 (39.0%), and aorto-bi-iliac grafts with concomitant hypogastric artery revascularisation (performed via jump grafting to preserve pelvic perfusion)—unilateral in 16 cases (8.2%) and bilateral in 6 (3.1%)—for a total of 22 cases (11.3%). IMA reimplantation or visceral reconstruction was performed in 91 cases (46.7%); renal artery reconstruction was performed in 18 (9.2%).

Median operative time was 261 min (IQR 210–320), reflecting systematic teaching involvement. Median intraoperative blood loss was 1250 mL (IQR 800–1900). Intraoperative cell salvage was routinely employed as institutional standard in all non-infected cases; allogeneic transfusion was reserved for emergency cases or when haemoglobin fell below 80 g/L despite cell salvage. Mean renal ischaemia time in juxtarenal/suprarenal cases was 33.5 min (SD 16.1) (Table 2). The median operative time of 261 min (IQR 210–320) is longer than would be expected in a purely senior-surgeon series, explicitly reflecting the time invested in structured intraoperative training.

### 3.3. Perioperative and Follow-Up Outcomes

The 30-day all-cause mortality was 1.5% (3/195). For elective cases (*n* = 162), 30-day mortality was 1.2% (2/162); for urgent or emergency cases (*n* = 33), it was 3.0% (1/33). To characterise the highest-acuity subgroup, we analysed the 17 ruptured AAAs separately. Thirty-day mortality in the ruptured cohort was 0% (0/17), 95% CI 0–19.5%, compared with 1.2% (2/162) in elective and 6.3% (1/16) in symptomatic non-ruptured cases (*p* = 1.000, ruptured vs. elective). Major morbidity in the ruptured cohort comprised acute kidney injury in 11.8% (2/17), pulmonary complications in 17.6% (3/17), colonic ischaemia in 0% (0/17), and perioperative reintervention in 5.9% (1/17); overall mortality at follow-up was 11.8% (2/17). None of these outcomes differed significantly between the ruptured, symptomatic, and elective groups (all *p* > 0.20).

Overall, no perioperative strokes occurred; myocardial infarction occurred in three patients (1.5%). Cardiac arrhythmia or failure was recorded in 12 patients (6.2%). Pulmonary complications were the most frequent adverse event (30 patients, 15.4%), consistent with the expected respiratory morbidity in an elderly comorbid population undergoing major abdominal surgery.

Acute kidney injury by KDIGO criteria occurred in 20 patients (10.3%); 30-day clinically significant renal insufficiency occurred in 10 patients (5.1%). Importantly, no patient required permanent renal replacement therapy. These rates are noteworthy given that 33.8% of cases required suprarenal or transrenal clamping.

Colonic ischaemia was identified in five patients (2.6%), of which four occurred in elective cases and one in the urgent/emergency setting; limb ischaemia occurred in six (3.1%) and spinal cord ischaemia in two (1.0%). Wound-related complications included evisceration in 3 patients (1.5%) and incisional hernia in 18 (9.2%). Prosthesis infection occurred in three patients (1.5%), all managed with explantation and reconstruction with bovine pericardial conduit.

Acute (perioperative) reinterventions occurred in 15 patients (7.7%), comprising limb ischaemia or graft thromboembolism (5), colonic ischaemia management (5), evisceration repair (3), compartment syndrome decompression (3), haemorrhage control (2), anastomotic revision (2), wound revision or haematoma drainage (3), and urological intervention (1). The three cases of prosthesis explantation included in our cohort were performed for late prosthetic graft infection following prior AAA repair and represented urgent admissions, not perioperative complications of the index procedure. Elective late reinterventions occurred in 20 patients (10.3%), principally incisional hernia repair (18 cases). Considering both groups together, the overall reintervention rate over the full follow-up period was 38/195 (19.5%); however, the clinically meaningful perioperative reintervention rate was 7.7%.

**Figure 3:**
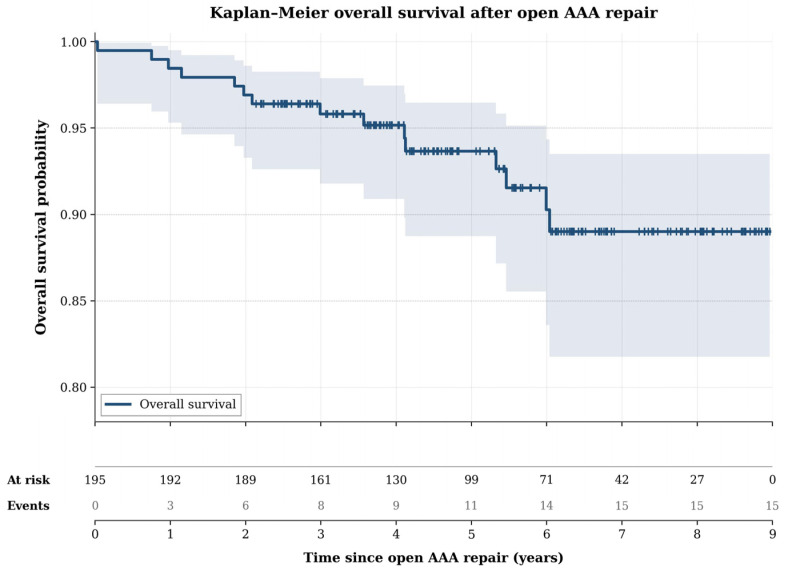
Kaplan–Meier estimates of overall survival in the 195 patients. Shaded area represents 95% confidence interval. Vertical tick marks represent censored patients. Estimated survival was 98.5% at 1 year, 96.9% at 2 years, 95.8% at 3 years, 95.1% at 4 years, 93.6% at 5 years, and 89.0% at 7 years. Numbers at risk are reported below the *x*-axis. Median follow-up was 61 months (IQR 44–81); follow-up was censored at 31 December 2025.

Median hospital stay was 8 days (IQR 7–12; mean 10.6 days, SD 7.8). Mean follow-up was 62.3 months (SD 25.1; median 61, IQR 44–81) over a 7-year enrolment period. Seven patients (3.6%) were lost to follow-up after the 30-day primary endpoint and were censored at their last known contact. Overall mortality was 15/195 (7.7%), with aneurysm-related mortality of 2 patients (1.0%). Kaplan–Meier estimated overall survival was 98.5% at 1 year, 96.9% at 2 years, 95.8% at 3 years, 95.1% at 4 years, 93.6% at 5 years, 90.3% at 6 years, and 89.0% at 7 years (Figure 3; Table 3 and Table 4).

## 4. Discussion

This series of 195 consecutive open AAA repairs demonstrates that perioperative outcomes benchmarking favourably against published high-volume centre data are achievable at a small- to medium-volume institution. The 30-day elective mortality of 1.2%, the absence of perioperative stroke, a low myocardial infarction rate (1.5%), and AKI rates at the lower end of published ranges collectively challenge the premise that institutional volume alone determines surgical quality. Moreover, the long-term survival profile—89.0% at 7 years on Kaplan–Meier estimation—confirms durable mid-term outcomes in this elderly, comorbid cohort.

### 4.1. The Open Approach at Our Centre

At our centre, endovascular procedures (EVAR, FEVAR, and BEVAR) collectively account for 52.2% of all aortic interventions performed during the study period. This endovascular proportion is lower than the 70–80% reported in several international registries and reflects a deliberate institutional strategy rather than limited endovascular capability. In fact, EVAR is offered predominantly to patients meeting anatomical instructions-for-use (IFU) criteria, whereas open repair is preferred in younger, lower-risk patients with a long life expectancy, as well as in cases with hostile or IFU-unsuitable neck anatomy, extensive iliac involvement, or connective-tissue disorders, in accordance with current ESVS guidelines [1]. In addition, the open approach is chosen when it facilitates single-stage management of concomitant aneurysmal disease, such as in patients with associated iliac or common femoral artery aneurysms. This selective approach is intended to prioritize long-term durability and freedom from reintervention over short-term perioperative advantages in patients expected to outlive the durability window of endovascular repair. These considerations govern elective decision-making; allocation in the emergency setting follows a different algorithm, in which endovascular repair is preferred whenever technically applicable (Section 2.1).

### 4.2. The Centralisation Paradigm and Its Limitations

Large registry analyses—including the German nationwide study by Trenner et al. covering over 96,000 AAA cases—have demonstrated a significant inverse association between hospital volume and in-hospital mortality, with the lowest mortality observed in hospitals performing more than 30 AAA procedures per year [4]. These data have strongly supported centralisation policies. However, the same registry acknowledges that volume is likely a surrogate for latent constructs such as team experience, infrastructure, and individual operator skill, and that very high-volume hospitals—typically receiving transferred high-risk patients—may paradoxically show a flattening of the volume-outcome curve [4].

Liang et al. provided a more granular analysis, demonstrating that both hospital and individual surgeon volume independently predict mortality after open AAA repair, with surgeon volume emerging as the dominant determinant [7]. Under their classification (low ≤ 2, medium 3–9, and high > 9 cases per surgeon per year), our individual surgeons fall within the medium- to high-volume category, even though our institution operates at medium institutional volume. Our outcomes are consistent with what this stratification predicts: medium institutional volume in the hands of experienced surgeons can yield results comparable to high-volume centres. We acknowledge that the boundaries between volume categories are not absolute: our mean of 27.9 cases per year lies close to the >30 cases/year threshold that Trenner et al. identified as the highest-volume quartile, and the concentration of the entire caseload in three surgeons places each at approximately nine open repairs per year—at or above the >9 cases/surgeon threshold that Liang et al. classify as high individual-surgeon volume. This is precisely the point of our study: an institution that is, by hospital-volume terminology, small to medium can nonetheless achieve high individual-surgeon volume and high-volume outcomes. Rather than contradicting our premise, this reinforces our central argument that surgeon volume and team performance, not institutional case counts, are the operative determinants of quality; we have clarified this framing to avoid any implication that our centre is low-volume at the surgeon level.

In large academic centres, an institutional annual volume of 20–30 open aortic repairs is typically distributed across multiple consultants, residents, and fellows, yielding an effective individual surgeon volume of as few as five to eight cases per year [7,8]. In contrast, at our centre, the entire open aortic caseload is concentrated in three senior surgeons with extensive prior experience at high-volume academic institutions.

### 4.3. Beyond the Surgeon: The Hospital System as a Determinant of Outcomes

While individual surgeon experience is a primary driver of outcomes, open aortic surgery is fundamentally a team-dependent endeavour. At our centre, operative indications are discussed within a structured multidisciplinary vascular board [9], ensuring that only appropriately selected patients proceed to open repair—a selection process that itself contributes to favourable outcomes independent of technical surgical skill. The perioperative anaesthetic team plays an equally critical role. Our anaesthesiologists manage a broad high-acuity surgical caseload, maintaining competence in major haemorrhage management, complex coagulation, and goal-directed haemodynamic therapy. A dedicated aortic anaesthesiologist is assigned to elective cases, ensuring familiarity with the specific physiological challenges of aortic cross-clamping, unclamping, and renal protection. The favourable AKI rate in our series—10.3% overall, with only 5.1% reaching clinical significance and no patient requiring permanent renal replacement—likely reflects the combined contribution of surgical renal protection protocols and anaesthetic management, rather than surgical technique alone.

Our data therefore support the concept that favourable outcomes in open aortic surgery reflect a ‘hospital system effect’—the aggregate performance of surgical, anaesthetic, nursing, and intensive care teams operating within a structured perioperative protocol—rather than the performance of the operating surgeon in isolation. Trenner et al. explicitly acknowledge this in their German registry analysis, noting that high-volume centres benefit from a ‘team routine effect’ that extends beyond individual operator volume [4]. Our results suggest this team-based quality can be replicated at moderate institutional volume when each individual team component maintains high standards. The outcomes of the 17 ruptured AAAs in our series are reported descriptively rather than as evidence of this hospital-system effect. Because the subgroup includes only patients who survived to aortic clamping, and because it comprises those ruptures in which endovascular repair was not feasible, no inference regarding the centre’s capacity to manage unselected rupture can be drawn from it (Section 4.6). Our argument therefore rests on the elective and symptomatic cohorts, in which the denominator is complete and the case mix is characterised.

### 4.4. Surgical Teaching and Outcome Preservation

One aspect of open aortic surgery practice that has received limited attention in the quality literature is the role of supervised surgical training [10]. Approximately 95% of our cases include a training component, with chief residents and senior registrars performing defined operative phases under the immediate, scrubbed supervision of a senior surgeon. The median operative time of 261 min reflects this teaching investment and is longer than the 180–220 min typically reported in purely senior-surgeon series.

Critically, this teaching engagement did not translate into worse outcomes; our 30-day elective mortality, complication rates, and renal outcomes are at or below benchmarks published from high-volume centres where trainee involvement is not systematically reported. Trenner et al. note that patients operated on by surgeons in training do not have worse outcomes after AAA repair when adequately supervised [4], a finding our data corroborate. This is consistent with evidence from other complex surgical disciplines [11,12]. We acknowledge an inherent tension in our position. Our senior surgeons acquired their expertise through extensive prior training at high-volume academic centres, and a strategy of complete decentralisation could, if applied uncritically, erode the very high-volume environments in which future surgeons must be trained. Our argument is therefore not against centralisation of training but against the conflation of institutional volume with quality when assessing established, experienced teams. A sustainable model likely requires a two-tier structure: concentration of complex-aortic training and the highest-acuity referrals in high-volume academic hubs, coupled with recognition that experienced surgeons who then practise within competent multidisciplinary teams at moderate-volume centres can safely maintain open aortic surgery. In this view, high-volume centres remain essential as training engines, while quality assessment of individual centres should rest on surgeon volume, team performance, and demonstrated outcomes rather than institutional case counts alone.

### 4.5. Benchmarking Against Published Standards

The SVS quality benchmark for elective open AAA repair is a perioperative mortality of ≤5% [2]. The German nationwide registry reported an overall OSR mortality of 5.3% across all volume groups, with only the highest-volume quartile (>30 cases/year) achieving 4.5% [4]. Our elective 30-day mortality of 1.2% substantially outperforms these benchmarks, despite a high-risk cohort: 15.9% ASA class IV, 27.7% juxtarenal anatomy, and 22.1% suprarenal clamping. The complete absence of perioperative stroke and a myocardial infarction rate of 1.5% across 195 cases compare favourably with the German registry, which reports stroke and myocardial infarction rates of 0.7% and 1.7%, respectively, even in the highest-volume hospitals [4]. Our AKI rate of 10.3% compares favourably with published series reporting rates of 15–30% for cohorts with comparable proportions of suprarenal and juxtarenal clamping [13], consistent with our protocolised renal protection strategy.

### 4.6. Limitations

This study has several limitations. It is a single-centre retrospective analysis with a modest sample size, limiting subgroup analytical power and precluding robust multivariable outcome analysis. The anaesthetic team is involved in surgical and interventional procedures of varying complexity across multiple specialties, and a potential selection bias in case allocation cannot be excluded. The absence of a concurrent comparator group from a high-volume centre prevents direct causal inference. The contribution of multidisciplinary team factors—anaesthetic management, nursing care, and intensive care expertise—to the observed outcomes cannot be formally quantified from these data, and our results likely reflect a collective team performance rather than surgical skill alone. Finally, our results reflect the practice of a single centre with a stable multidisciplinary team and may not be universally reproducible.

A specific limitation concerns the ruptured subgroup. The 30-day mortality of 0% (0/17) after open repair of ruptured AAA is a substantial outlier relative to contemporary international benchmarks of 30–50%, and we do not present it as an expected or reproducible outcome for unselected ruptured AAA populations. The estimate is statistically fragile: with 17 patients, the exact binomial 95% confidence interval extends to approximately 19.5%, and a single additional death would have shifted the point estimate to 5.9%. The subgroup is also selected at two levels. First, only patients who survived transfer and reached the operating theatre are included, whereas those who died in the emergency department or during transport and those turned down for surgery were not captured by a database constructed around operated cases (Section 2.1); the institutional intention-to-treat mortality for ruptured AAA is therefore unknown from these data and certainly higher. Our referral area includes several remote districts from which timely transfer may be difficult, and the most unstable patients are consequently liable to be under-represented in an operated cohort. Second, the study is restricted to open repair, whereas endovascular repair is preferred at our institution whenever technically applicable, so these 17 patients represent the ruptures in which it was not feasible rather than all ruptures reaching theatre. Admission haemodynamic variables were not available for the present analysis, and the proportion of free intraperitoneal ruptures with profound shock versus contained retroperitoneal leaks cannot be established here. This subgroup is therefore reported for descriptive completeness only; the observed result most plausibly reflects small sample size and patient selection rather than a reproducible standard of rupture management, and no conclusion regarding institutional performance in aortic rupture should be drawn from it.

## 5. Conclusions

This consecutive series of 195 open AAA repairs demonstrates that favourable perioperative and mid-term outcomes—including a 30-day elective mortality of 1.2%, no perioperative strokes, a low myocardial infarction rate (1.5%), and a Kaplan–Meier 7-year overall survival of 89.0%—are achievable at a small- to medium-volume centre. These results reflect the synergistic contribution of individual surgeon expertise, structured supervised teaching, rigorous operative technique, and a high-functioning multidisciplinary perioperative team.

We advocate for a more nuanced approach to quality assessment in open aortic surgery—one that evaluates individual surgeon volume, team competence, and demonstrated outcomes, rather than institutional case counts alone. Centralisation policies that rely exclusively on institutional volume thresholds risk conflating size with quality, potentially denying patients access to experienced surgical teams operating at moderate-volume institutions with favourable track records.

## Figures and Tables

**Table 1:** Patient demographics and baseline characteristics (*n* = 195).

Characteristic/Variable	Value (*n* = 195)
Demographics	
Male sex, *n* (%)	167 (85.6)
Age, years, mean (SD)	70.2 (8.1)
Age, years, median (IQR)	70 (65–76)
Age range, years	40–88
BMI, kg/m^2^, mean (SD)	26.1 (4.1)
Comorbidities	
Hypertension, *n* (%)	142 (72.8)
Smoking, current or former, *n* (%)	104 (53.3)
Coronary heart disease, *n* (%)	58 (29.7)
Chronic obstructive pulmonary disease, *n* (%)	39 (20.0)
Diabetes mellitus, *n* (%)	36 (18.5)
Congestive heart failure, *n* (%)	24 (12.3)
Previous open abdominal surgery, *n* (%)	29 (14.9)
ASA Physical Status	
ASA class II, *n* (%)	34 (17.4)
ASA class III, *n* (%)	129 (66.2)
ASA class IV, *n* (%)	31 (15.9)
Renal Function—KDIGO staging *	
CKD stage 1 (GFR ≥ 90 mL/min/1.73 m^2^), *n* (%)	36 (25.4%)
CKD stage 2 (GFR 60–89), *n* (%)	102 (71.8%)
CKD stage 4 (GFR 15–29), *n* (%)	3 (2.1%)
CKD stage 5 (GFR < 15), *n* (%)	1 (0.7%)

ASA, American Society of Anesthesiologists; BMI, body mass index; CKD, chronic kidney disease; GFR, glomerular filtration rate; IQR, interquartile range; KDIGO, Kidney Disease: Improving Global Outcomes; SD, standard deviation. * KDIGO percentages are calculated on the 142 patients with available pre-operative renal function staging.

**Table 2:** Aneurysm characteristics and operative data (*n* = 195).

Characteristic/Variable	Value (*n* = 195)
Aneurysm Characteristics	
Diameter, mm, mean (SD)	60.0 (12.9)
Diameter, mm, median (IQR)	56 (54–62)
Diameter range, mm	33–120
Asymptomatic presentation, *n* (%)	162 (83.1)
Symptomatic presentation, *n* (%)	16 (8.2)
Ruptured AAA, *n* (%)	17 (8.7)
Fusiform morphology, *n* (%)	139 (71.3)
Operative Setting	
Elective repair, *n* (%)	162 (83.1)
Urgent or emergency repair, *n* (%)	33 (16.9)
Surgical Approach	
Transperitoneal, *n* (%)	184 (94.4)
Retroperitoneal (lombotomy), *n* (%)	11 (5.6)
Aortic Clamping Level	
Infrarenal, *n* (%)	128 (65.6)
Transrenal, *n* (%)	24 (12.3)
Suprarenal, *n* (%)	43 (22.1)
Reconstruction Type	
Straight tube graft, *n* (%)	97 (49.7)
Aorto-bi-iliac bifurcated graft, *n* (%)	76 (39.0)
Aorto-bi-iliac with unilateral hypogastric jump graft, *n* (%)	16 (8.2)
Aorto-bi-iliac with bilateral hypogastric jump graft, *n* (%)	6 (3.1)
Total with hypogastric revascularisation (jump grafting), *n* (%)	22 (11.3)
Adjunctive Procedures	
IMA reimplantation or visceral reconstruction, *n* (%)	91 (46.7)
Renal artery reconstruction (cases requiring it), *n* (%)	18 (9.2)
Intraoperative Data	
Operative time, min, median (IQR) *	261 (210–320)
Operative time, min, mean (SD) *	273.4 (92.2)
Blood loss, mL, median (IQR)	1250 (800–1900)
Blood loss, mL, mean (SD)	1517 (1172)
Renal ischaemia time, min, mean (SD) †	33.5 (16.1)
Intraoperative cell salvage	Routinely employed ‡

AAA, abdominal aortic aneurysm; IMA, inferior mesenteric artery; IQR, interquartile range; SD, standard deviation. * Operative time includes supervised teaching cases (Section 2.2). A training component was present in approximately 95% of cases, which directly contextualises these values. † Renal ischaemia time measured in cases with juxtarenal or suprarenal clamping. ‡ Intraoperative cell salvage was used as standard institutional protocol in all non-infected cases; allogeneic transfusion was reserved for emergency cases or when haemoglobin fell below 80 g/L despite cell salvage.

**Table 3:** Perioperative and follow-up outcomes (*n* = 195).

Outcome	*n* (%)
Mortality	
30-day all-cause mortality (*n* = 195)	3 (1.5)
30-day mortality—elective cases (*n* = 162)	2 (1.2)
30-day mortality—urgent/emergency cases (*n* = 33)	1 (3.0)
Overall mortality at follow-up	15 (7.7)
Aneurysm-related mortality	2 (1.0)
Cardiac and Neurological Events	
Stroke	0 (0)
Myocardial infarction	3 (1.5)
Cardiac failure/arrhythmia	12 (6.2)
Systemic Complications	
Pulmonary complications	30 (15.4)
Urinary complications	11 (5.6)
Delirium	6 (3.1)
Renal Outcomes	
Acute kidney injury (KDIGO any stage)	20 (10.3)
30-day clinically significant renal insufficiency	10 (5.1)
Permanent renal replacement therapy	0 (0)
Vascular Complications	
Colonic ischaemia (4 elective, 1 urgent)	5 (2.6)
Limb ischaemia	6 (3.1)
Spinal cord ischaemia	2 (1.0)
Wound and Prosthesis Complications	
Evisceration	3 (1.5)
Incisional hernia	18 (9.2)
Prosthesis infection	3 (1.5)
Reinterventions	
Acute reinterventions (perioperative)	18 (9.2)
Limb ischaemia/graft thromboembolism	5 (2.6)
Colonic ischaemia (incl. Hartmann)	5 (2.6)
Evisceration repair	3 (1.5)
Prosthesis explantation for infection	3 (1.5)
Compartment decompression	3 (1.5)
Haemorrhage/haemostasis	2 (1.0)
Anastomotic/endoluminal revision	2 (1.0)
Urological intervention	1 (0.5)
Elective late reinterventions	20 (10.3)
Incisional hernia repair	18 (9.2)
Other elective late procedures	2 (1.0)
Overall reintervention rate (all causes)	38 (19.5)
Hospital Stay and Follow-up	
Length of hospital stay, days, median (IQR)	8 (7–12)
Length of hospital stay, days, mean (SD)	10.6 (7.8)
Follow-up duration, months, mean (SD)	62.3 (25.1)
Follow-up duration, months, median (IQR)	61 (44–81)

AKI, acute kidney injury; IQR, interquartile range; KDIGO, Kidney Disease: Improving Global Outcomes; SD, standard deviation. The acute (perioperative) reintervention rate was 9.2%; the overall rate of 19.5% includes 18 elective incisional hernia repairs performed during long-term follow-up.

**Table 4:** Elective vs. urgent/emergency subgroup comparison.

Variable	Elective (*n* = 162)	Urgent (*n* = 33)	*p* Value
Age, years, median (IQR)	70 (65–76)	74 (64–79)	0.379
Aneurysm diameter, mm, median (IQR)	56 (54–60)	69 (53–80)	0.037
Operative time, min, median (IQR)	268 (210–321)	250 (188–306)	0.312
Blood loss, ml, median (IQR)	1300 (800–1900)	1000 (625–1975)	0.350
Length of stay, days, median (IQR)	8 (7–10)	9 (7–15)	0.303
30-day mortality, *n* (%)	2 (1.2)	1 (3.0)	0.430
AKI any stage, *n* (%)	16 (9.9)	4 (12.1)	0.751
Pulmonary complications, *n* (%)	25 (15.4)	5 (15.2)	1.000

Continuous variables compared with Mann–Whitney U test; categorical variables with Fisher’s exact test. AKI, acute kidney injury; IQR, interquartile range. *p* < 0.05 considered statistically significant. As expected, aneurysm diameter was significantly larger in the urgent/emergency subgroup.

## Data Availability

Aggregate data are available upon reasonable request.

## Abbreviations

The following abbreviations are used in this manuscript:

AAA	Abdominal Aortic Aneurysm
AKI	Acute Kidney Injury
ASA	American Society of Anesthesiologists
BMI	Body Mass Index
CKD	Chronic Kidney Disease
COPD	Chronic Obstructive Pulmonary Disease
BEVAR	Branched Endovascular Aortic Repair
ESVS	European Society for Vascular and Endovascular Surgery
EVAR	Endovascular Aortic Repair
FEVAR	Fenestrated Endovascular Aortic Repair
GenAI	Generative Artificial Intelligence
IFU	Instructions-For-Use
IMA	Inferior Mesenteric Artery
OSR	Open Surgical Repair
SVS	Society for Vascular Surgery
IQR	Interquartile Range
KDIGO	Kidney Disease: Improving Global Outcomes
MACCE	Major Adverse Cardiovascular and Cerebrovascular Events
REBOA	Endovascular aortic balloon occlusion
SD	Standard Deviation
